# The BET family in immunity and disease

**DOI:** 10.1038/s41392-020-00384-4

**Published:** 2021-01-19

**Authors:** Nian Wang, Runliu Wu, Daolin Tang, Rui Kang

**Affiliations:** Department of Surgery, UT Southwestern Medical Center, Dallas, TX 75390 USA

**Keywords:** Infection, Diseases

## Abstract

Innate immunity serves as the rapid and first-line defense against invading pathogens, and this process can be regulated at various levels, including epigenetic mechanisms. The bromodomain and extraterminal domain (BET) family of proteins consists of four conserved mammalian members (BRD2, BRD3, BRD4, and BRDT) that regulate the expression of many immunity-associated genes and pathways. In particular, in response to infection and sterile inflammation, abnormally expressed or dysfunctional BETs are involved in the activation of pattern recognition receptor (e.g., TLR, NLR, and CGAS) pathways, thereby linking chromatin machinery to innate immunity under disease or pathological conditions. Mechanistically, the BET family controls the transcription of a wide range of proinflammatory and immunoregulatory genes by recognizing acetylated histones (mainly H3 and H4) and recruiting transcription factors (e.g., RELA) and transcription elongation complex (e.g., P-TEFb) to the chromatin, thereby promoting the phosphorylation of RNA polymerase II and subsequent transcription initiation and elongation. This review covers the accumulating data about the roles of the BET family in innate immunity, and discusses the attractive prospect of manipulating the BET family as a new treatment for disease.

## Introduction

The immune system is composed of special organs, cells, and chemicals that can prevent various infections (e.g., from bacteria and viruses) and injuries (e.g., wounds and trauma) by activating innate and adaptive immune responses.^1^ Unlike adaptive immunity, in which immune cells (e.g., B and T cells) target specific antigens through the recognition by either antibodies or cell receptors, innate immunity mediated by myeloid cells (e.g., neutrophils, monocytes, macrophages, and dendritic cells [DCs]) and natural killer (NK) cells is rapid and antigen-independent.^2^ Defects in or the excessive activation of the innate immune system may lead to inflammation, which is implicated in various diseases and pathological conditions, such as cancer^3^ diabetes,^4^ and sepsis.^5^ This process is strictly regulated at the epigenetic, transcriptional, posttranscriptional, and posttranslational levels.

Epigenetic changes in immune cells are a key component of gene activation during the inflammatory response, which causes the production of immune mediators (e.g., cytokines and chemokines) and the infiltration, polarization, or re-population of immune cells.^6^ The epigenetic mechanisms have many forms, such as DNA modification (e.g., methylation and oxidation), posttranslational modification of histones (e.g., acetylation, methylation, phosphorylation, ubiquitylation, and SUMOylation), nucleosome positioning, and changes in microRNA (miRNA) expression.^7^ Among them, histone acetylation is a reversible chromatin modification mediated by histone acetyltransferases (HATs, also called “writers”) and histone deacetylases (HDACs, also termed as “erasers”). Furthermore, acetyl-binding proteins (namely “readers”) mainly recognize acetylated histones.^8^ Abnormal changes in epigenetic readers modify gene expression and disrupt the cellular machinery, thereby changing the function of immune cells.

Bromodomain is an evolutionarily conserved protein-protein interaction module consisting of approximately 110 amino acids that can recognize and bind acetylated lysine residues in histones and many other proteins.^9^ Bromodomain-containing proteins (BRDs) serve as epigenetic readers of histone acetylation, which can recruit transcriptional regulator complexes to chromatin and bind to acetylated histones.^10^ In 2012, 61 bromodomain modules were identified among 46 different proteins in the human genome, and these BRDs were divided into 8 subfamilies (e.g., bromodomain and extraterminal domain [BET] subfamilies) based on the similarity of protein sequences.^11^ The BET family contains four related proteins (namely BRD2, BRD3, BRD4, and BRDT) that act as epigenetic readers with broad specificity on transcriptional activation (including the recruitment of positive transcription elongation factor [P-TEFb] and the control of RNA polymerase II [Pol II] transcriptional activity).^12^ A dysfunctional BET family member is involved in many physiological and pathological processes and has become an important therapeutic target for diseases, including immune and inflammatory diseases.^13^

In this review, we summarize the emerging role of the BET family in innate immunity and highlight its functions in various diseases through orchestrating pattern recognition receptor (PRR) signaling and transcriptional regulation of immune genes. We also discuss the potential application of BET inhibitors (BETis) in regulating immune homeostasis in diseases, and then look forward to future research directions in this area.

### Classification and structure of BETs

**Fig. 1:**
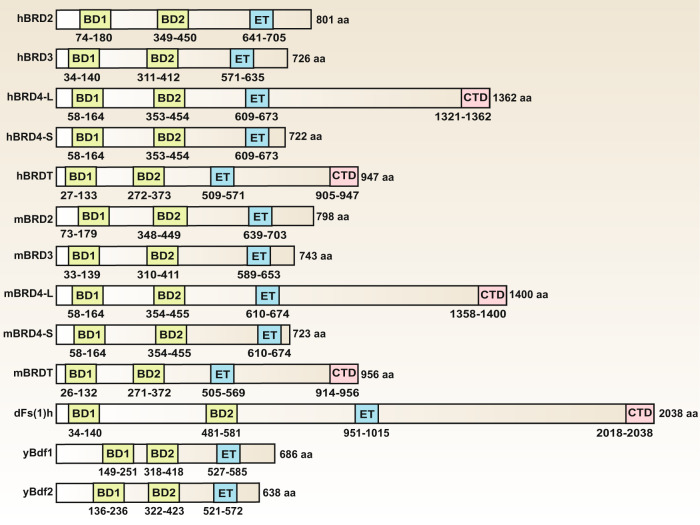
Domain architecture of BET family proteins in human (h), mouse (m), *Drosophila* (d), and yeast (y). Numbers indicate the amino acid boundaries of each domain in individual proteins. Alignment of amino acid sequences was based on published information using the following accession numbers retrieved from GenBank databases: hBRD2, NM_005104; hBRD3, NM_007371.4; hBRD4-L, NM_058243; hBRD4-S, NM_014299; hBRDT, NM_001242805; mBRD2, NM_010238; mBRD3, NM_001113574; mBRD4-L, NM_020508; mBRD4-S, NM_198094; mBRDT, NM_014299; dfs(1)h, NM_078523; yBdf1, NM_001182287; yBdf2, AJV11655. Abbreviations: BD1, the first bromodomain; BD2, the second bromodomain; Bdf1, chromatin-binding protein BDF1; BRD, bromodomain protein (numbers 2–4); CTD, C-terminal domain; ET, extraterminal domain; Fs(1)h, female sterile (1) homeotic

In mammalian cells, four BETs with similar gene arrangements, domain organizations, and functions have been identified.^14^ In humans, these genes are referred to as BRD2 (also known as FSRG1, RING3, RNF3, FSH, or D6S113E), BRD3 (also known as ORFX or RING3L), BRD4 (also known as MCAP or HUNK1), and BRDT (also known as BRD6, CT9, or SPGF21). In mice, these genes are designated as Brd2 (also known as Frg1, Fsrg1, Nat, Ring3, or Rnf3), Brd3 (also known as Fsrg2, Orfx, or Ringl3), Brd4 (also known as Fsrg4 or Mcap/Hunk1), and Brdt (also known as Fsrg3 or Brd6). Both human and mouse BRD4 have long and short isoforms, and the relative abundance of these two forms of BRD4 vary among different cell types.^15,16^ The long isoform of BRD4 (BRD4L) is a well-characterized coactivator of transcription (corresponding to the ordinary full-length transcript), whereas the short isoform of BRD4 (BRD4S) corresponds to an alternative splicing variant lacking exons 12–20.^16^ Although BRD4L and BRD4S have the same N-terminal conserved tandem bromodomains and extraterminal domain, they are not functionally redundant because the opposite roles of these two isoforms have been found in different contexts.^17^ Mammalian BETs are highly conserved and have homologs in other species, such as Fs(1)h in *Drosophila*, and BDF1 and BDF2 proteins in *Saccharomyces cerevisiae*, but these homologs are not fully understood (Fig. 1).

Each BET protein is characterized by the presence of two N-terminal conserved tandem bromodomains (namely the first bromodomain [BD1] and the second bromodomain [BD2]) and a unique extraterminal (ET) domain in the C-terminal moiety.^14^ Other protein families containing bromodomains lack this double-barrel feature. Other domains, such as motif B and Ser/Glu/Asp-rich region (SEED), are also highly conserved in BETs, whereas the C-terminal domain (CTD) and motif A are not present in each protein. The BD structure contains four alpha helices, which are separated by a variable loop region, thus forming a hydrophobic cavity. Acetylated lysine can be recognized by this central hydrophobic pocket via anchoring to a conserved asparagine residue. Of note, BETs prefer to bind to di-acetylated lysine residues closely located in the protein sequence, which is distinguished from other BRDs.^11,18^ Although the amino acid residues critical for binding acetyl-lysine in BD1 and BD2 are highly conserved, low homology is found between these two domains; thus they independently regulate the expression of BET-sensitive genes.^19^ However, BD1 and BD2 exhibit >75% identity with the homologous domains in different BETs.

In addition to acetylated lysine in histones, BETs also interact with transcription factors (TFs) and transcription elongation complexes (e.g., P-TEFb) through lysine acetylation-dependent or -independent mechanisms.^20^ The interaction of BDs with acetylated chromatin either at gene promoters or in long range *cis* regulatory elements (namely “enhancers”) allows subsequent initiation of gene transcription.^21^ The C-terminal ET domain is responsible for additional protein-protein interactions, thus enabling BETs as scaffolds for the recruitment of TFs and coactivators. BETs also regulate gene transcription through their intrinsic kinase activities because the regions they possess are weakly reminiscent of kinase motifs.^22,23^ However, these kinase motifs lack homology with other known kinase domains, and the biochemical mechanisms of the kinase activities remain elusive.

### Expression of BETs in innate immunity

BRD2, BRD3, and BRD4 are significantly expressed in the nucleus, indicating that these proteins play a major role in regulating DNA events. Ordinarily, BRD2 is highly expressed in pancreatic β cells, germ cells in testis and ovaries, neurons in the cerebellum and cerebral cortex, liver, spleen, lungs, and kidney.^24,25^ BRD3 is commonly expressed in testis, ovaries, placenta, uterus, endocrine tissue, adipose tissue, lungs, kidney, muscle, and skin.^11^ BRD4 is more ubiquitously expressed and most abundant in bone marrow and lymphoid, mid-gestation embryos, testis, ovaries, adipose tissue, kidneys, and brain, whereas BRDT is selectively presented in the testis and ovaries.^26^ Although different BETs can be found in the same type of tissue, they are usually distributed distinctly from each other and exert different functions, suggesting that BETs are not simply redundant.^27^ Notably, BRD2, BRD3, and BRD4 are abnormally expressed in activated immune cells (e.g., macrophages and NK cells) as described below.

### BRD4

**Fig. 2:**
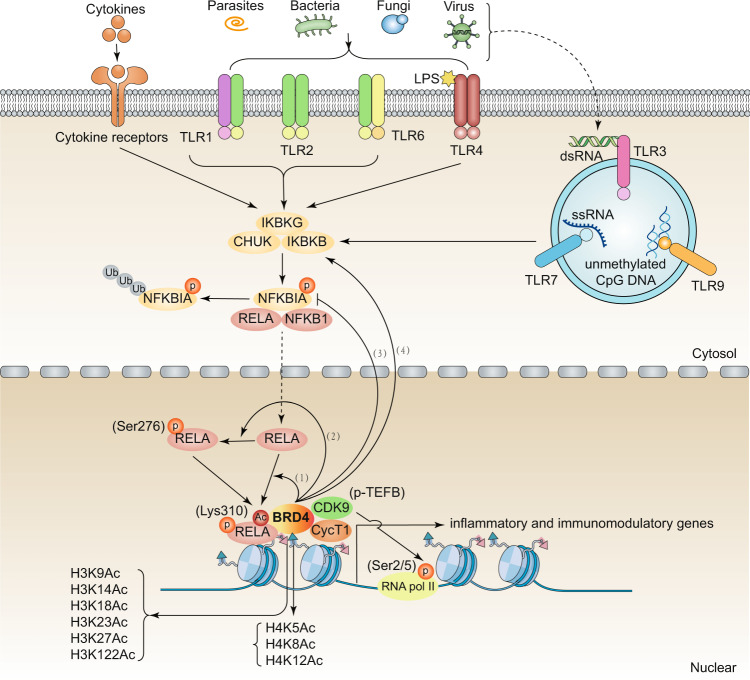
The role BRD4 in regulating the NF-κB pathway in inflammation and immunity. BRD4 regulates the activation of the NF-κB pathway caused by TLR ligands through at least four pathways: (1) BRD4 directly acetylates RELA through its atypical histone acetyltransferase activity; (2) BRD4 directly promotes the phosphorylation of RELA; and (3–4) BRD4 promotes the phosphorylation of RELA through inhibiting the translation of NFKBIA and increasing the phosphorylation of NFKBIA and IKBKB. Abbreviations: CDK9, cyclin-dependent kinase 9; CHUK, component of inhibitor of nuclear factor kappa B kinase complex; CycT1, cyclin T1; IKBKB, inhibitor of nuclear factor kappa B kinase regulatory subunit beta; IKBKG, inhibitor of nuclear factor kappa B kinase regulatory subunit gamma; NFKBIA, NFKB inhibitor alpha; NFKB1, nuclear factor kappa B subunit 1; p-TEFb, positive transcription elongation factor b; RELA, RELA proto-oncogene; RNA pol II, RNA polymerase II; TLR, toll-like receptor (numbers 1–9)

BRD4 is the most well-studied BET protein in response to various stresses, including infection and immune stimulation (Table 1 and Fig. 2). BRD4 expression is markedly upregulated in different resident immune cells (e.g., macrophages, monocytes, T cells, and NK cells) and non-immune cells (e.g., pulmonary microvascular endothelial cells, bronchial epithelials, cardiomyocytes, and smooth muscle cells) under various stimuli (e.g., cigarette smoke extract, viruses, and listeriolysin-O).^28–30^ In these cells, BRD4 mainly exerts proinflammatory roles through conferring transcription activation of a variety of immune and inflammatory genes, and it may serve as a detrimental stress protein that can be used to predict disease activity. Moreover, the upregulation of BRD4 is also found in the uterus and fetal membranes induced by labor and infection,^31^ and severe early-onset preeclampsia placenta,^32^ which may lead to adverse pregnancy outcomes. In addition to normal cells, BRD4 is highly expressed in different types of tumor cells (e.g., cells of renal cell carcinoma,^33^ malignant pleural mesothelioma,^34^ pancreatic ductal adenocarcinoma,^35^ melanoma,^36^ and primary human mucoepidermoid carcinoma^37^), and it can protect these tumor cells against immunogenic cell death, a type of regulated cell death that enhances tumor targeting immunity. These findings indicate that BRD4 acts as a mediator of tumorigenesis and as a powerful prognostic biomarker. However, in some rare cases (e.g., in memory CD4+ T cells in HIV-1–infected individuals), BRD4 expression is downregulated, which may be a consequence of increased immune activation because of the opposing effect on expression induced by interferon (IFN) in viremic HIV-1 infection.^38^ It is also worthy to note that BRD4 function is not completedly determined by the change of its expression, as it also occupies essential roles in various pathophysiological processes with no detectable change in its expression, such as in pseudorabies virus infection, chronic obstructive pulmonary disease, and spinal cord injury.^39–41^ The expression of BRD4 changes inversely in different types of cells even in the same disease state. Hence, the immunomodulatory roles of BRD4 are highly cell-type dependent, which is regulated by unknown molecular switches.

**Table 1:** Mechanism and function of BRDs in immunity and disease

BET	Expression	Disease model	Cell type	Treatment	Transcription factor	Binding sites in histones	Target genes	BETi	Refs
**Infectious diseases**									
***Bacterial infection***									
BRD2 BRD4	N/A	Gram-negative bacterial infection	Mouse SIM-A9 microglial cell line, mouse primary astrocytes	LPS	N/A	N/A	NOS2, COX2, IL1B, TNF, IL6, CCL2, MMP9, SERPINE1	dBET1, JQ1	^46,131^
BRD2 BRD3 BRD4	N/A	Endotoxemia	Mouse bone marrow derived macrophages (BMDMs)	LPS	RELA, IRF4, IRF8	H3Ac, H4K5Ac, H4K8Ac, H4K12Ac, H4Ac	TNF, IL6, IFNB1, CCL2, IL1B, IL12A, CXCL9, CCL12	I-BET, JQ1	^69^
BRD4	N/A	Sepsis, group B *Streptococcus* (GBS) infection	Mouse BMDMs	GBS, LPS, cecal ligation puncture	RELA	N/A	IL6, IL1A, IL12B, CXCL9, IL23A	N/A	^97^
BRD2 BRD4	Upregulation	*Pseudomonas aeruginos* infection	Human bronchial epithelial cells	IL17	N/A	N/A	IL17A, IL22, CXCL1, CXCL5, CXCL8, CCL2, CSF3, IL6	CPI-203	^186^
BRD2 BRD3 BRD4	N/A	Heat-killed *L. monocytogenes* infection	Mouse BMDMs	IFNB	RELA	N/A	NOS2, IL6, IL1RN	JQ1	^94^
***Virus infection***									
BRD4	Upregulation	RNA virus (respiratory syncytial virus infection)	Human small airway epithelial cells, nonciliated secretoglobin (Scgb1a1)-expressing bronchiolar epithelial cells	Poly(I:C)	RELA, IRF1, IRF7, IRF3	H3K122Ac	IL6, ISG54, CIG5, RANTES, FN1, COL1A, MMP9, VIM, ACTA2, HEXIM1, KC, CSF3, CSF2, CXCL1, CXCL2, CXCL8, CCL2	ZL0513, ZL0516, ZL0420, ZL0454, JQ1	^55–58,110,187^
BRD4	No change	Virus infection	Pig PK15 cells	Pseudorabies virus, herpes simplex virus, ectromelia virus, etc.	IRF3, RELA	H3K9, H3K27, H4K8, H4K12, H4K16	IL1B, IFNB, ISG15	JQ1, OTX-015, I-BET 151	^39^
BRD4	Downregulation	HIV-1 infection	Memory CD4+ T cells	N/A	N/A	N/A	N/A	N/A	^38^
BRD4	N/A	HIV-1 infection	J-Lat A2 cell line	N/A	N/A	N/A	HIV	JQ1	^107^
BRD3	Downregulation	Virus infection	Mouse macrophage cell line RAW 264.7	Sendai virus, vesicular stomatitis virus, herpes simplex virus	IRF3	H3/H4	IFNB1	N/A	^50^
***Fungi and parasitic infection***									
BRD2 BRD3 BRD4	N/A	*Candida albicans* and *Aspergillus fumigatus* infection	Whole blood cells, peripheral blood mononuclear cells, monocytes	N/A	N/A	H3K4Me3, H3K27Ac	TNF, IL6	I-BET151, JQ1	^129^
BRD2	Upregulation	*Plasmodium yoelii* and *Toxoplasma gondii*-infected liver	N/A	N/A	N/A	N/A	TNF, IFNG	N/A	^140^
BRD2 BRD3 BRD4	N/A	*Schistosoma japonicum* infection	Human Th17 cells	N/A	RORC	N/A	IL17, IL21, CSF2	JQ1	^141^
**Non-infectious diseases**									
***Cancer***									
BRD4	Upregulation	Renal cancer	Human renal cell carcinoma cell lines	LPS	RELA	H3K27Ac	CXCL1, CXCL8, CXCR2, CSF2, CSF3, NLRP3	JQ1	^33,148^
BRD4	N/A	Prostate cancer	Human prostate cancer cell lines (DU145, PC3)	N/A	N/A	N/A	CD274, HLA-A, HLA-C, IFNG	JQ1	^152^
BRD4	N/A	Pancreatic cancer	Pancreatic cancer cell lines (PANC-1, BxPC-3, MIA PaCa-2), pancreatic stellate cells	IFNG	IRF1	N/A	CD274	JQ1, IBET	^153,154^
BRD2 BRD3 BRD4	N/A	Pancreatic cancer	Aspc-1, PANC-1, CAPAN-1 cells, mouse pancreatic cancer cell lines PanAsc 2159 and Panc 1343, RAW 264.7	IFNG, LPS	STAT3	N/A	IL6, CCL2, CSF2	JQ1, I-BET 762	^35,102^
BRD3	N/A	Gastric adenocarcinoma	Microsatellite instability high gastric cancer cells	N/A	N/A	N/A	CD274	N/A	^46^
BRD2 BRD3 BRD4	N/A	A549 tumor-bearing nude mice, neuroblastoma	N/A	N/A	N/A	N/A	CD274	JQ1	^155^
BRD4	N/A	Triple-negative breast cancer, hepatocellular carcinoma	Peripheral blood mononuclear cells, monocytes	N/A	CEBPB	H3K27Ac	CD274	JQ1, I-BET762	^115,156^
BRD4	N/A	Triple-negative breast cancer	Human breast cancer cell line	IL6	N/A	N/A	JAG1	JQ1	^147^
BRD2 BRD4	Upregulation	Malignant pleural mesothelioma	Human primary malignant pleural mesothelioma cells	N/A	N/A	N/A	CD274, PDCD1	JQ1, OTX015	^34^
BRD2	N/A	Melanoma	Human melanoma cell lines (Mel-RMu, SK-Mel28, Mel-RM, Mel-JD, Me1007)	N/A	RELA	N/A	IL6, IL8, VEGF, CXCL10, RANTES	I-BET151	^87^
BRD2 BRD3 BRD4	N/A	Human Ty-82 xenografts	Ty-82, SKOV3, A549, MDA-MB-231 cells	N/A	N/A	H3K27Ac	IDO1	ABBV-075, JQ1, OTX015	^126^
BRD4	Upregulation	Breast cancer with T-bet+ tumor-infiltrating T lymphocytes	T-bet+ TILs	N/A	N/A	N/A	JAG1	N/A	^149^
BRD4	Upregulation	Mucoepidermoid carcinoma	Human mucoepidermoid carcinoma cells	N/A	RELA	N/A	N/A	I-BET762	^37^
BRD2 BRD3 BRD4	N/A	Neuroblastoma	NK cells	N/A	MYC, TP53	N/A	ULBP1, ULBP3, PVR, NECTIN2	JQ1	^188^
BRD2 BRD3 BRD4	N/A	Primary effusion lymphoma	N/A	N/A	RELA	N/A	IL6	JQ1	^189^
BRD2 BRD3 BRD4	N/A	Myeloproliferative neoplasms	JAK2V617F-positive SET-2 cells	N/A	RELA	H3K27Ac	CCL2, CCL3, CCL4, CCL5, IL10, IL6, IL13, CXCL9, CSF3, II15, CXCL10, II1A, CXCL2, CXCL5	JQ1	^61^
BRD4	N/A	Multiple myeloma	Human myeloma cell lines (SKO-007(J3), U266, ARP-1, RPMI-8226), human multiple myeloma cell line (JJN-3)	N/A	MYC	N/A	MICA	ARV-825 (PROTAC), JQ1, I-BET151	^133^
***Cardiovascular diseases***									
BRD4	Upregulation	Pathological cardiac hypertrophy	Neonatal mouse cardiomyocytes	Aortic banding, angiotensin-II	RELA	N/A	TNF, IL1B	N/A	^158^
BRD4	Upregulation	Pulmonary arterial hypertension	Human microvascular endothelial cells, smooth muscle cells	N/A	FOXM1	N/A	FOXM1, PLK1, IL8, MCP1, CCL5	RVX208	^159^
BRD2 BRD3 BRD4	N/A	Pulmonary arterial hypertension	Human pulmonary microvascular endothelial cells	N/A	RELA	N/A	IL6, IL8	JQ1	^62^
BRD2 BRD3 BRD4	N/A	Cardiovascular disease	Human monocytic cell line (THP-1), human umbilical vein vessel endothelial cells, human artery endothelial cells	TNF, LPS, IL1B	RELA	N/A	VCAM1, CCL2, IL8, SELE, IL1B	Apabetalone, MZ-1	^132^
BRD2 BRD3 BRD4	N/A	Vascular inflammation	Human umbilical vein vessel endothelial cells	TNF, LPS	RELA	N/A	ICAM1, VCAM1, SELE	JQ1	^13^
BRD4	Upregulation	Hypertension	Rat vascular smooth muscle cells	Angiotensin II	JUN, RELA, STAT1, CDX2, FOXL1, LIN54, ETS1	H3K27Ac	ESM1, SPRY2, TGIF1, FST, FGF2, EGR2, FLT, FOXP1	JQ1	^190^
BRD2 BRD3 BRD4	N/A	Heart failure (prolonged pressure overload, massive anterior myocardial infarction)	Human induced pluripotent stem cell-derived cardiomyocytes	ET1	RELA, JUN, STAT1	N/A	NPPB, CTGF	JQ1	^101^
BRD2 BRD4	Upregulation	Acute myocardial infarction	N/A	N/A	N/A	N/A	TLR4, TRAF6, RELA, CRP, IL6	JQ1	^66^
BRD4	N/A	Atherogenesis	Human umbilical vein vessel endothelial cells	TNF	RELA	H3K27Ac	VCAM1, SOX18, CCL2	JQ1	^63^
***Respiratory diseases***									
BRD4	Upregulation	Chronic obstructive pulmonary disorder	Human pulmonary microvascular endothelial cells	Cigarette smoke extract	N/A	N/A	IL6, IL8, TNF	N/A	^29,30^
BRD2 BRD3 BRD4	No change	Chronic obstructive pulmonary disorder	Alveolar macrophages	LPS	N/A	N/A	IL6, CSF2	JQ1	^40^
BRD4	N/A	Asthma	Primary human small airway epithelial cells	Cat dander extract	RELA	H3K122Ac	COL1, FN1, ZEB1, SNAI1, VIM, IL6	ZL0454	^163^
BRD2 BRD3 BRD4	N/A	Asthma	CD4+ T cells, human bronchial epithelial cells	Cockroach allergen extract	N/A	N/A	IL4, IL17A, RORC, IL23R	JQ1	^191^
BRD4	N/A	Asthma	Asthmatic airway smooth muscle cells	FCS, TGFB	N/A	N/A	IL6, IL8	JQ1, I-BET762	^192^
BRD2 BRD3 BRD4	N/A	Neutrophil-dominant allergic airway disease	CD4 + CD62L + naïve T cells	IL23, TGFβ, IL6, anti-IL4, anti-IFNG	N/A	N/A	IL1A, IL1B, IL2, IL6, IL10, IL12B, IL13, IL17A, CCL11, CSF3, CXCL1, CCL4, CCL5	CPI-203	^193^
BRD4	N/A	Pulmonary fibrosis	Lung fibroblasts	Bleomycin	N/A	H4K5Ac	IL6	JQ1	^124^
***Neurological diseases***									
BRD2	Upregulation	Parkinson’s disease	Mouse primary microglial cells	Alpha-synuclein (αSynAgg)	STAT3	N/A	ACOD1, IFIT1, PYHIN, CDC123, SOD1, GRN	N/A	^44^
BRD2 BRD3 BRD4	N/A	Systemic sclerosis	Human monocytes of systemic sclerosis patients	N/A	STAT1, STAT2, IRF	H3K27Ac	MX1, CMPK2	JQ1	^109^
BRD4	Upregulation	Cerebral ischemia/reperfusion injury	Mouse astrocytes, microglial BV2 cells	Oxygen-glucose deprivation/reperfusion	RELA	N/A	IL6, IL1B, IL18, TNF	JQ1	^75^
BRD2 BRD3 BRD4	N/A	Spinal cord injury	BMDMs	LPS	N/A	N/A	IL6, IL1B, TNF, IL4, IL13	JQ1	^166^
BRD2 BRD3 BRD4	No change	Spinal cord injury	Primary cerebellar granule neurons, astrocytes, oligodendrocytes, microglias	IL1B, TNF	N/A	N/A	IL6, IL1B, CCL5, CCL2, CXCL10, TNF	JQ1	^41^
BRD2	Upregulation	Neuronal damage after deep hypothermic circulatory arrest	Mouse microglial BV2 cells, human neuroblastoma SH-SY5Y cells	Oxygen-glucose deprivation	RELA	N/A	TNF, IL5, IL10, IL13	JQ1	^88^
BRD2 BRD3 BRD4	N/A	Autoimmune encephalomyelitis	N/A	N/A	N/A	N/A	IL6, IL17, CCL2, CSF2, IFNG, TNF	RVX-297	^194^
BRD2 BRD3 BRD4	N/A	Alzheimer’s disease	N/A	N/A	N/A	N/A	IL1B, IL6, TNF, CCL2, NOS2, PTGS2	JQ1	^165^
***Kidney diseases***									
BRD4	N/A	Experimental renal damage	Human renal proximal tubular epithelial cells (HK2)	Nephrotoxic serum, TNF	RELA	N/A	IL6, CCL2, CCL5	JQ1	^95^
BRD2 BRD3 BRD4	N/A	Stage 4 or 5 chronic kidney disease	N/A	N/A	N/A	N/A	IL6, PAI1, OPN	Apabetalone	^14^
BRD4	Upregulation	Lupus nephritis	N/A	N/A	N/A	N/A	IL1B, IL6, IL17, IL10, INFG	JQ1	^168^
BRD4	N/A	HIV-associated kidney disease	Human primary renal tubular epithelial cells	TNF, HIV	RELA	H3K9Ac, H3K18Ac, H3K4me3, H3K27me3	IL1A, IL1B, LTA, LTB, CCL2, CCL3, CCL20, CXCL3, CXCL11, CCL2, CCL20, IL8	MS417	^167^
***Digestive diseases***									
BRD4	Upregulation	Acute liver injury	Mouse Kupffer cells	Listeriolysin-O	RELA		TNF, IL6, IL1B, IL18, CCL2, CCL8	JQ1	^136^
BRD4	N/A	Colitis	Mouse intestinal epithelial cells	Dextran sulfate sodium	RELA	H3K9Ac	TNF, IL1B	N/A	^169^
BRD4	N/A	Colitis	Th17 cells	N/A	N/A	H4K5Ac, H4K8Ac	IL17, IFNG, IL21, IL22, RORC, TBX21, IL6, GATA3	MS402	^20^
BRD2 BRD3 BRD4	N/A	Colitis	Mouse BMDCs, human monocyte-derived DCs	LPS	N/A	N/A	IL6, IL12B, IL10	I-BET151	^195^
BRD2 BRD3 BRD4	N/A	Non-alcoholic fatty liver disease, liver fibrosis	N/A	N/A	N/A	N/A	IFNG, CCL2, TNF, RSAD2, LY6A, CD4, CD7, CXCL10, STAT1, CCL5	I-BET151	^100^
BRD2 BRD3 BRD4	N/A	Acute pancreatitis	N/A	N/A	N/A	N/A	IL6, IL10, CCL2, CXCL1	I-BET762	^170^
***Metabolic diseases***									
BRD2 BRD4	N/A	Cancer cachexia	N/A	N/A	FOXO3	N/A	IL6, TNF, IL1B, PTHLH	JQ1	^174^
BRD2 BRD4	N/A	Obesity, insulin resistance, diabetes	INS-1 cells, mouse adipocytes (3T3-L1)	TNF	PPARG, RELA	N/A	INS, TNF, CXCL2, IL1B, IL6, CCL2	JQ1	^24,89,172^
BRD2 BRD3 BRD4	N/A	Type 1 diabetes	Pancreatic macrophages, β cells	LPS	RELA	N/A	CCL2, CCL5, CXCL1, CXCL2, IFNB1, IL1A, IL1B, NFKBIA, TNF, TNFAIP3, TNFRSF9, VCAM1, CXCL9, IL12B, IL6, MX1, MX2, RSAD2	I-BET151	^171^
***Osteoarthritis***									
BRD4	Upregulation	Intervertebral disc degeneration	Nucleus pulposus cells	TNF, advanced glycation end products	RELA	N/A	NLRP3, CASP1, MMP13	JQ1	^28,89^
BRD4	N/A	Acute gouty arthritis	Human monocytic cell line (THP-1)	Monosodium urate	RELA	N/A	IL1B	Benzo[cd]indol-2(1H)-ones, Pyrrolo[4,3,2- dequinolin-2(1H)-ones	^74^
BRD4	Upregulation	Periprosthetic osteolysis	Mouse macrophage RAW264.7 cells	Titanium particles	RELA	N/A	TNF, IL1B, IL6	JQ1	^196^
BRD3 BRD4	N/A	Osteoarthritis	Human chondrosarcoma cells (SW1353)	IL1B, TNF		H4K5Ac, H4K8Ac, H4K12Ac	MMP1, MMP3, MMP13, ADAMTS4	I-BET151	^118^
BRD2 BRD3 BRD4	N/A	Failure of bone healing	C2C12 and MC3T3-E1 cell lines, BMDMs	TNF	RUNX2, SP7	N/A	ALP, RUNX2, SP7	N-methylpyrrolidone and N, N-dimethylacetamide, JQ1	^197^
BRD4	Upregulation	Articular cartilage of osteoarthritis	Human chondrocyte cell line sw1353 cells	IL1B	RELA	H3K27Ac	HMGB1, IL1B	JQ1	^176^
BRD2 BRD3 BRD4	Upregulation	Rheumatoid arthritis	Human rheumatoid arthritis fibroblast-like synoviocytes	TNF, IL1B	JUN, RELA	N/A	TNF, IL1B, IL6, IL8, IL17, IL18, MMP1, MMP3, MMP13	JQ1, I-BET151	^51,98,177^
***Others***									
BRD2 BRD3 BRD4	Upregulation	Spontaneous preterm birth	Myometrial cells, amnion epithelial and mesenchymal cells	LPS, IL1B	RELA	N/A	IL6, CCL2, CXCL1, CXCL8	JQ1	^31^
BRD4	Upregulation	Preeclampsia	Primary trophoblasts, human umbilical vein vessel endothelial cells	TNF	N/A	N/A	IL6, CXCL8, CCL2, CXCL1, FLT1	N/A	^32^
BRD4	N/A	Autoimmune uveitis (EAU)	Human CD4 + T cells, human Th17-polarized cells	Anti-CD3/CD28	RORC	N/A	IL17A, IL22, RORC, IL10	GSK151, JQ1	^178^
BRD2	Upregulation	Inherited retinal degeneration	Mouse primary microglias	LPS	N/A	N/A	TNF, CCL2, IL1B, IL6, RANTES	JQ1, RVX-208	^45^
BRD2 BRD3 BRD4	N/A	Age-related macular degeneration	Human retinal pigment epithelial cell line (ARPE-19)	Etoposide	RELA	N/A	IL6, IL8	JQ1, PFI-1, I-BET151	^179^
BRD2	N/A	Psoriasis	Primary human keratinocytes, HS27 dermal fibroblasts	IL17A, TNF, IL22	RELA		IL8, CXCL1, CXCL2, CXCL6, CXCL8, GCSF, CCL20	JQ1, I-BET151	^198^
BRD2 BRD3 BRD4	N/A	Psoriasis-like inflammation	N/A	Imiquimod	RORC	N/A	IL17A, IL22	JQ1	^180^
BRD4	N/A	Periodontitis	RAW264.7 cells	LPS	RELA	N/A	IL1B, IL6, TNF, TLR2, TLR4	JQ1	^51^

Although the regulatory mechanism controlling BRD4 expression is not clear, different miRNAs act as posttranscriptional regulators of BRD4 expression under various conditions. For example, miR-218-5p, miR-29a, miR-29b, and miRNA-302e inhibit BRD4 expression in activated human pulmonary microvascular endothelial cells, primary hepatic stellate cells, human bronchial epithelial cells, and A549 cells, respectively.^30,42,43^ We still need to clarify the TFs responsible for BRD4 expression in innate immunity.

### BRD2

Compared with BRD4, less attention is paid to the expression of BRD2 and BRD3, which are also implicated in the regulation of immune response. Early studies showed that BRD2 is highly expressed in pancreatic β cells, thereby inhibiting mitosis and insulin transcription.^24^ Recent studies indicate that α-synuclein induces BRD2 expression, thus initiating the neuroinflammation in Parkinson’s disease.^44^ The expression of BRD2 (but not BRD3 and BRD4) is dominantly increased during photoreceptor degeneration that occurs postnatally in lipopolysaccharide (LPS)-stimulated mouse primary astrocytes.^45,46^ Elevated BRD2 expression is also detectable in malignant pleural mesothelioma, melanoma, and cardiomyocytes following acute myocardial infarction, which is concordant with BRD4.^45,46^ Moreover, the upregulation of BRD2 may be the main driver of LPS- or spinal cord injury-induced gene expression in macrophages or neuronal cells.^40,47^ Although the mechanism of BRD2 upregulation is unclear, it seems that BRD2 acts as a stress-sensitive metabolism-related protein after inflammatory stimulation.

### BRD3

While BRD3 and BRD2 have overlapping cellular functions, their expression patterns exhibit some differences. Frameshift mutations of BRD3 have been found in gastric cancer, which is negatively related to the expression of CD274 (also known as programmed death ligand 1 [PD-L1]), a transmembrane protein that downregulates immune responses.^48^ BRD3 expression is significantly downregulated during endothelial differentiation^49^ or virus infection in macrophages.^50^ In contrast, BRD3 expression is upregulated in activated lymphocytes, indicating a potential role of BRD3 in adaptive immunity.^49^ BRD3 is also detected in the macrophages in synovial tissues derived from rheumatoid arthritis patients and osteoarthritis patients, which is similar to BRD2 and BRD4 expression,^51^ suggesting that different BETs may have a synergistic effect in autoimmune diseases. Notably, BRD2 and BRD3 can exert roles with no detectable change in their expressions.^41^ Therefore, both the expression and structure of BETs occupy essential roles in the regulation of gene expression and immune response. More in-depth studies are needed to clarify the regulatory mechanisms of their expression in the future.

### Function of BETs in innate immunity

The innate immune response is mainly triggered by the recognition of various extracellular or intracellular danger signals through PRRs expressed in immune and non-immune cells.^52^ The surface-expressed PRRs include toll-like receptors (TLRs), C-type lectin receptors (CLRs), and advanced glycosylation end-product specific receptors (AGER/RAGE), whereas the intracellular PRRs include nucleotide-binding oligomerization domain (NOD)-like receptors (NLRs), retinoic acid-inducible gene (RIG) I-like receptors (RLRs), AIM2-like receptors (ALRs), and cyclic GMP-AMP synthase (CGAS). These PRRs directly or indirectly recognize the evolutionary conservative structure on pathogens (namely pathogen-associated molecular patterns [PAMPs], such as microbial nucleic acids, LPS, and carbohydrates) or endogenous molecules (namely damage-associated molecular patterns [DAMPs], such as high-mobility group box 1 [HMGB1], histones, host nucleic acids, and ATP). In response to PAMPs or DAMPs, gene transcription is activated and precisely controlled by TFs and coactivators coupled to BETs.

### BETs in TLR signaling

#### TLR3

TLR3, a sensor of viral infections, is preferentially activated by dsRNA derived from the extracellular RNA viral genome, and it triggers the production of type I IFNs.^53^ TLR3 is mainly expressed in hematopoietic cells, particularly in a subset of DCs, but is also expressed in some stromal cells. The specificity of TLR3 for dsRNA allows its recognition of various RNA viruses, such as respiratory syncytial virus (RSV), influenza A virus, West Nile virus, and rhinovirus.^54^ Thus, the long-term activation of TLR3 is implicated in various respiratory diseases.

BETs link the TLR3 signaling pathway to chromatin remodeling and specific inflammatory gene transcription. BRD4 seems to play a major role in TLR3-induced acute airway inflammation and remodeling, and specific BETis have been developed to inhibit this process. BRD4 mediates poly(I:C)-induced airway inflammation by promoting the transcription of CIG5, IL6, KC, CCL2, ORM2, CXCL2, IFNB, ISG54, and CCL5 in airway epithelial cells or lung tissues and further increases the secretion of inflammatory cytokines in bronchoalveolar lavage fluid.^55^ Selective BRD4 inhibitor targeting of BD1 obviously alleviates inflammatory response by reversing cytokine expression.^56,57^ RSV also induces neutrophilic inflammation and the production of chemokines (CSF2, CXCL2, IL8, CCL2, and CCL5) and mucosal IFN in the nonciliated SCGB1A1-expressing epithelium through the binding of RELA/p65 to BRD4.^58^ In addition to BRD4, BRD3 promotes the production of type I IFN in macrophages during vesicular stomatitis virus infection.^50^ I-BET151 (a pan-BETi) suppresses the expression of cytokines (IL6 and IL8) and MMP3 in rheumatoid arthritis synovial fibroblasts in response to poly(I:C).^51^ In addition, I-BET151 inhibits the association of BRD4 with interferon beta 1 (IFNB1) promoter, thereby reducing IFNB1-mediated gene transcription in macrophages following poly(I:C) or LPS stimulation.^59^ These findings suggest that BRD4 is essential for TLR3-stimulated activation of the IFNB1 pathway and its antitumor activity.

#### TLR4

TLR4 is expressed in almost all innate immune cells, and it specifically recognizes bacterial LPS, several other components of pathogens, and DAMPs derived from tissue damage.^60^ TLR4 binds to its ligands, which ultimately leads to the activation of the nuclear factor kappa light-chain enhancer of activated B cells (NF-κB) signaling pathway and subsequently the production of inflammatory cytokines involved in innate immune response. RELA (also known as p65) is a member of the NF-κB family and the main subunit of NF-κB transcription factor complex. BETs are coactivators of RELA (discussed separately below), which is recruited to the promoter of target genes and enhances their transcriptional activation.^61–64^ In addition to mediating RELA activation, BETs positively regulate TLR4 expression through different molecular mechanisms. For example, in pancreatic cancer cell lines (PANC-1 and BxPC-3), BRD4 promotes the expression of TLR4 through the transcriptional activation of CD276 (also known as B7-H3), which supports the role of the BRD4-TLR4 pathway in the regulation of immunotherapy and chemotherapy in pancreatic cancer.^65^ JQ1 (a pan-BETi) significantly reduces the protein expression of TLR4, TRAF6, and NF-κB in the heart of rats with acute myocardial infarction.^66^ Moreover, JQ1 reduces the expression of TLR4 and the production of inflammatory cytokines (e.g., IL1B, IL6, TNF, and IL10) in LPS-induced macrophages.^67^ Overall, these findings indicate that BRD4 is implicated in both infectious and sterile inflammation through controlling TLR4 expression and activation.

#### TLR1, TLR2, and TLR6

TLR2 in association with TLR1 or TLR6 is implicated in the recognition of a wide range of components (e.g., di- and triacylated lipoproteins and lipoteichoic acid) from Gram-positive or -negative bacteria.^68^ Although the effect of BETs on these TLRs has not been fully explored, JQ1 may reduce the expression of TLR2, which is proposed to be implicated in the production of inflammatory cytokines in diseased gingival tissues.^67^ In addition, in LPS-induced bone marrow-derived macrophages, the BETi (e.g., I-BET) reduces *Tlr6* mRNA expression to 2.3 folds, but the precise mechanism and pathological role of this downregulation has not been investigated.^69^ It is unclear whether the genetic depletion of BET protein has a similar effect to that of BETis in the regulation of TLR2 and TLR6 expression as well as their activation-mediated innate immune responses.

#### TLR7 and TLR9

TLR9 mainly recognizes unmethylated CpG motifs, which are abundant in bacterial or viral DNA.^70^ Unlike TLR9, TLR7 is an endosomal innate immune sensor used to detect single-stranded ribonucleic acid.^71^ Both TLR7 and TLR9 stimulation facilitate the production of type I IFNs and other inflammatory cytokines, which greatly contribute to the antiviral immune responses. While there is no direct evidence that BETs regulate the expression of TLR7 and TLR9, the roles of BETs in the production of IFNs have been demonstrated. In particular, in the human plasmacytoid DC line Gen2.2, different pan-BETis (e.g., JQ1 and I-BET151) block the production of IFNB caused by TLR7 or TLR9 agonists (e.g., CL097 and ODN1826).^59^ BRD3 is also found to promote the transcription of IFNB1 in macrophages during vesicular stomatitis virus, sendai virus, or herpes simplex virus infection.^50^ These findings provide a line of evidence indicating that BETs link TLR7 and TLR9 activation to mount inflammation and immune responses through the transcription activation of IFNs. It will be interesting to learn whether BETs also regulate TLR8-, TLR10- and TLR11-dependent signaling pathways in innate immunity.

#### BETs in NLR signaling

NLRs are cytoplasmic PPRs that can recognize PAMPs and DAMPs, and they also play a crucial role in initiating the innate immune response. According to the structure of the N-terminal domain, NLRs can be divided into four subfamilies, namely NLRA, NLRB, NLRC, and NLRP.^72^ The NLRP subfamily contains NLRP1 through NLRP14, which are involved in the formation of inflammasomes, multiprotein oligomers of the innate immune system that are responsible for the activation of inflammatory responses.^73^ Among the NLRPs, NLRP3 is the most studied and best characterized inflammasome, which is triggered by various inflammatory stimuli. NLRP3 inflammasome activation further promotes caspase 1 (CASP1) or caspase 11 (CASP11) activation, leading to the induction of pyroptosis and the release of proinflammatory cytokines (e.g., IL1B and IL18) and DAMPs (e.g., HMGB1).

**Fig. 3:**
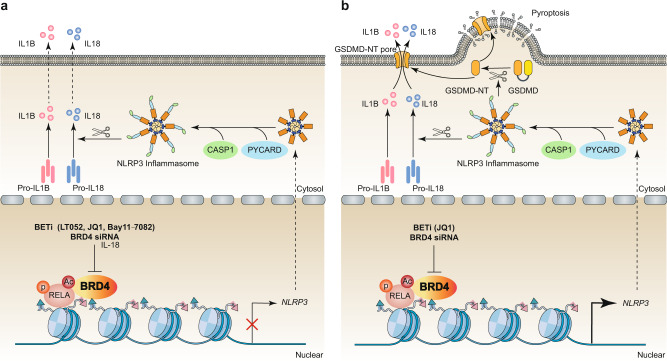
The role BRD4 in regulating the NLRP3 pathway in inflammation and pyroptosis. **a** In TNF-primed rat nucleus pulposus cells, in which monosodium urate induced acute gouty arthritis, and middle cerebral artery occlusion induced glial activation, BRD4 inhibits RELA-mediated NLRP3 transcription and subsequent CASP1-dependent inflammasome activation. **b** In renal cell carcinoma tissues and cells, BRD4 is required for RELA-mediated NLRP3 transcription and subsequent CASP1-dependent inflammasome activation and GSDMD-mediated pyroptosis. Abbreviations: BETi, bromodomain and extraterminal inhibitor; BRD4, bromodomain containing 4; CASP1, caspase 1; GSDMD, gasdermin D; GSDMD-NT, gasdermin D N-terminal domain; IL1B, interleukin 1 beta; IL18, interleukin 18; NLRP3, NLR family pyrin domain containing 3; PYCARD, PYD and CARD domain containing; RELA, RELA proto-oncogene; TNF, tumor necrosis factor

The pharmacological or genetic inhibition of BRD4 alleviates inflammatory response by inhibiting NLRP3 signaling pathways in varous conditions, such as in TNF-primed rat nucleus pulposus cells,^28^ monosodium urate-induced acute gouty arthritis,^74^ and middle cerebral artery occlusion-mediated glial activation.^75^ Mechanistically, BRD4 inhibition decreases the expression of NLRP3 and CASP1 by limiting the transcriptional activity of RELA. Of note, in different situations, BETs may have an effect that is opposite to that of limiting NLRP3 inflammasome activation. For instance, in renal cell carcinoma tissues and cells, the inhibition of BRD4 through genetic knockdown or JQ1 prevents proliferation and epithelial mesenchymal transition (EMT) by increasing RELA-mediated NLRP3 expression and subsequent pyroptosis.^33^ Collectively, the role of BETs in NLRP3 inflammasomes is cell type-specific, which depends on different transduction signals (Fig. 3). There remain challenges to indentifying whether BETs involved in orchestrating other inflammasomes (e.g., NLRP1, NLRC4, and absent in melanoma 2 [AIM2])-mediated immune responses.

#### BETs in CGAS signaling

**Fig. 4:**
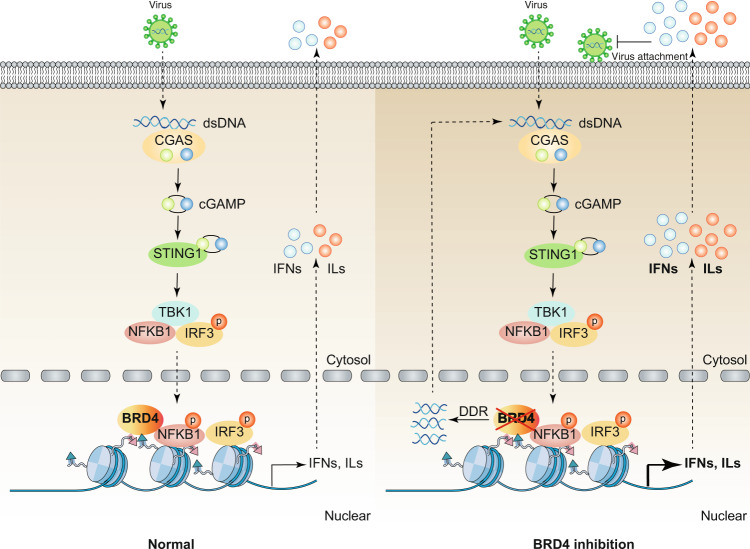
The role of BRD4 in regulating the STING1 pathway in antiviral immunity. Cytoplasmic DNA derived from various viruses activates CGAS and produces endogenous cyclic dinucleotide cGAMP, which binds to STING1 located in the endoplasmic reticulum, and then promotes the dimerization and translocation of STING1 from the ER to the perinuclear region. During trafficking, STING1 recruits and activates TBK1, stimulates the phosphorylation and nuclear translocation of IRF3, and to a lesser extent NFKB1, which leads to the production of type 1 IFN and other inflammatory cytokines (e.g., IL). The nuclear activity of IRF3 and NFKB1 is inhibited by BRD4. In addition, after BRD4 inhibition, the activation of DDR can induce the release of host dsDNA from the nucleus to the cytoplasm, leading to further activation of the CGAS-STING1 pathway to limit viral infection. Abbreviations: BRD4, bromodomain containing 4; cGAMP, cyclic GMP-AMP; CGAS, cyclic GMP-AMP synthase; DDR, DNA damage response; dsDNA, double-stranded DNA; IFN, interferon; IL, interleukin; IRF3, interferon regulatory factor 3; NFKB1, nuclear factor kappa B subunit 1; STING1, stimulator of interferon response cGAMP interactor 1; TBK1, TANK binding kinase 1

CGAS is a cytosolic DNA sensor that catalyzes the synthesis of cyclic dinucleotide cGMP-AMP (ultimately 2’3’-cGAMP) after activation.^76^ As a second messenger, 2’3’-cGAMP binds and activates an endoplasmic reticulum membrane adaptor protein, namely, stimulator of interferon response CGAMP interactor 1 (STING1, also known as STING or TMEM173) by inducing its conformational changes.^77^ The CGAS-STING1 pathway-mediated DNA-sensing signaling pathway is crucial for the production of type I IFNs and host antiviral responses.^78^ Interestingly, blocking BRD4 widely inhibits the attachment of various DNA and RNA viruses (e.g., pseudorabies virus) by activating nucleic acid-dependent antiviral innate immunity in vitro and in vivo. The role of BRD4 inhibition in antiviral immunity is partly mediated by the activation of the CGAS-STING1 pathway^39^ (Fig. 4). Excessive activation of the CGAS-STING1 pathway by bacterial cyclic dinucleotides causes cytokine storms and systemic coagulation, leading to sepsis and septic shock.^79^ During bacterial infections and DAMP-mediated sterile inflammation, it remains to be seen whether BRD4 and other BETs play a similar role in regulating the activation of the CGAS-STING1 pathway.

### Mechanism of BETs in innate immunity

**Fig. 5:**
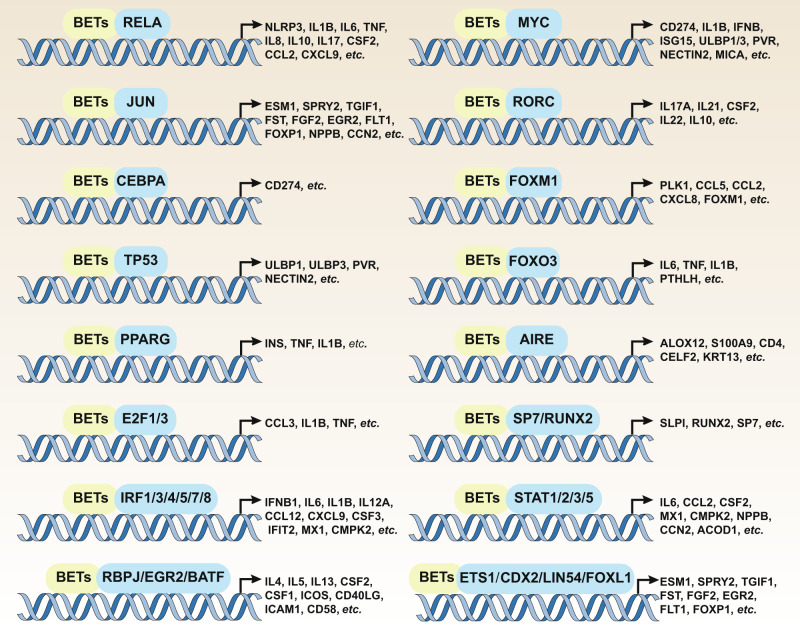
The role of BETs in regulating the expression of immune genes mediated by transcription factors. Abbreviations: ACOD1, aconitate decarboxylase 1; AIRE, autoimmune regulator; ALOX12, arachidonate 12-lipoxygenase, 12 S type; BATF, basic leucine zipper ATF-like transcription factor; CCL, C-C motif chemokine ligand (numbers 2–12); CCN2, cellular communication network factor 2; CD, CD molecule (numbers 4–274); CD40LG, CD40 ligand; CDX2, caudal-type homeobox 2; CEBPA, CCAAT enhancer binding protein alpha; CELF2, CUGBP Elav-like family member 2; CMPK2, cytidine/uridine monophosphate kinase 2; CSF1, colony stimulating factor (numbers 1–3); CXCL9, C-X-C motif chemokine ligand 9; E2F1, E2F transcription factor 1; EGR2, early growth response 2; ESM1, endothelial cell-specific molecule 1; ETS1, ETS proto-oncogene 1; FGF2, fibroblast growth factor 2; FLT1, fms-related receptor tyrosine kinase 1; FOXL1, forkhead box L1; FOXM1, forkhead box M1; FOXO3, forkhead box O3; FOXP1, forkhead box P1; FST, follistatin; ICAM1, intercellular adhesion molecule 1; ICOS, inducible T-cell costimulatory; IFIT2, interferon-induced protein with tetratricopeptide repeats 2; IFNB1, interferon beta 1; IL1B, interleukin 1 beta; IL, interleukin (numbers 4–22, including “A” variants); INS, insulin; IRF, interferon regulatory factor; JUN, Jun proto-oncogene, AP-1 transcription factor subunit; KRT13, keratin 13; LIN54, lin-54 DREAM MuvB core complex component; MX1, MX dynamin-like GTPase 1; MYC, MYC proto-oncogene, bHLH transcription factor; NECTIN2, nectin cell adhesion molecule 2; NLRP3, NLR family pyrin domain containing 3; NPPB, natriuretic peptide B; PLK1, polo-like kinase 1; PPARG, peroxisome proliferator activated receptor gamma; PTHLH, parathyroid hormone-like hormone; PVR, PVR cell adhesion molecule; RBPJ, recombination signal binding protein for immunoglobulin kappa J region; RELA, RELA proto-oncogene, NF-κB subunit; RORC, RAR-related orphan receptor C; RUNX2, RUNX family transcription factor 2; S100A9, S100 calcium-binding protein A9; SLPI, secretory leukocyte peptidase inhibitor; SP7, Sp7 transcription factor; SPRY2, sprouty RTK signaling antagonist 2; STAT, signal transducer and activator of transcription; TGIF1, TGFB-induced factor homeobox 1; TNF, tumor necrosis factor; TP53, tumor protein p53; ULBP1, UL16 binding protein (numbers 1–3)

As epigenetic reader proteins, BETs recognize and bind to acetylated lysine residues on histone tails, thereby facilitating the assembly of transcription complexes, including specific TFs, mediators, and RNA Pol II-mediated transcriptional initiation machinery. BET-mediated innate immune control mainly relies on the binding of BRD4 to RELA and the P-TEFb transcription extension complex to activate RNA Pol II. In addition to RELA, the BET-mediated immune response is also involved in the regulation of other TF activators, such as the signal transduction and transcription activation (STAT) protein family, the E2F transcription factor (E2F) family, and the interferon regulatory factor (IRF) family (Fig. 5). Below, we discuss how BETs regulate gene expression and innate immunity by affecting the activity and function of TFs, transcription coactivators, and histones.

### TFs

#### RELA

NF-κB pathways, including the RELA-dependent canonical pathway and RELB-dependent noncanonical pathway, is critical for the modulation of immune response.^80^ Under normal conditions, RELA is usually sequestered in the cytoplasm in an inactive form due to its binding to IκBα. Under inflammatory conditions, various PAMPs and DAMPs activate IκB kinases (IKKs), which leads to the phosphorylation, ubiquitination, and subsequent proteasomal degradation of IκBα. This dynamic modification and expression change of IκBα further promotes the transport of RELA from the cytoplasm to the nucleus, where it can bind to the promoters of various related genes and regulate their transcription.^81^ Both acetylation and phosphorylation are essential for the transcriptional activity of RELA.^82^ RELA acetylation is regulated by the acetyltransferase activity of CREB binding protein (CREBP)/E1A binding protein P300 (EP300) and lysine acetyltransferase 2B (KAT2B), and it occurs at multiple sites, including lysine (Lys)122, −123, −218, −221, and −310.^83,84^ Among these, the acetylation of Lys310 is required for the full transcriptional activity of RELA. BRD4 triggers the transcription activation of RELA by specifically binding to acetylated Lys310^85^. BRD4 deficiency or BETi also promotes the ubiquitination and degradation of the active nuclear form of RELA,^64,85^ indicating a dual role of BRD4 in the regulation of RELA. However, the regulation and mechanisms of BRD4 on the ubiquitination and degradation of RELA in immune cells remains mysterious. BRD2 also plays a role in promoting RELA activation in marcophages,^86^ melanoma,^87^ microglia,^88^ and adipose tissue,^89^ and the subsequent production of inflammatory mediators. Compared with BRD2, the mechanism of BRD4 in regulating the transcriptional activity of RELA has been extensively studies (Fig. 2), as described below.

#### BRD4 promotes RELA acetylation

Acetylated RELA is essential for recruiting BRD4 proteins to the promoters of target genes, which initiates inflammation, leading to cardiac fibrosis, myeloproliferative neoplasms, and airway inflammation.^61^ Increasing the binding between RELA and BRD4 can modify the chromatin environment and further promote the acetylation of RELA lysine 310 through BRD4’s atypical histone acetyltransferase activity, resulting in the transcriptional activation of inflammation and fibrosis genes (e.g., ACTA2, COL1A, FN1, MMP, IL6, KC, and neutrophilic chemokines).^55^ In addition, in human airway epithelial cells, hydrogen peroxide enhances the expression of IL6 and CXCL8 induced by IL1B through promoting the acetylation of RELA and the binding of BRD4 to the promoters of IL6 and CXCL8, while BRD2 has no effect on this process.^90^ Together, these findings indicate that there is a positive feedback between the formation of the RELA-BRD4 complex and the production of RELA acetylation, which is important for the transcriptional activity of RELA.

#### BRD4 promotes RELA phosphorylation

In addition to RELA acetylation, RELA phosphorylation contributes to the transcription activation of various inflammatory and catabolic genes. BRD4 promotes gene transcription by increasing the phosphorylation of RELA under various stimuli, such as IL1B, IL6, NOS2, and COX2 in highly aggressive proliferating immortalized (HAPI) microglia cells^91^ induced by LPS in rats. In mammalian cells, the cyclin-dependent kinase (CDK) family is the main regulator of the cell cycle and the initiator of DNA replication. The formation of the CDK9-BRD4 complex is required for RELA phosphorylation at Ser276-mediated RELA acetylation at Lys310, which leads to downstream inflammatory gene expression during RSV infection.^92^ Phospho-ser276 RELA is also required for the recruitment of the CDK9-BRD4 complex to core EMT transcriptional regulators (e.g., SNAI1, TWIST1, and ZEB1) to promote transforming growth factor beta 1 (TGFB1)-induced EMT.^93^ In macrophages infected with monocytogenes of the intracellular bacterial pathogen *Listeria*, two inhibitor of NF-κB kinase subunit beta, namely BI605906 and BETi JQ1, exhibit the same effect on the recruitment of BRD4 to NOS2 promoter and its expression, indicating that RELA activation is required for BRD4 binding to the target genes.^94^

Apart from recruiting phosphorylated RELA to the promoters of inflammatory genes, RELA can also form inflammatory superenhancers (SEs) and modulate the activities of global enhancer by altering the occupancy of BRD4. For example, canonical proinflammatory stimuli, such as TNF-α, enhance the binding of BRD4 to the SEs of proinflammatory genes in endothelial cells, thereby promoting the development of atherogenesis.^63^ JQ1-mediated inhibition of RELA phosphorylation-dependent cytokine production not only abrogates experimental renal inflammation in murine models,^95^ but also exhibits anti-inflammatory and anti-remodeling effects on human pulmonary microvascular endothelial cells.^62^ BRD4 and other BET members may play overlapping or different roles in regulating the expression of NF-κB-dependent inflammatory genes. For example, the knockdown of BRD2, BRD3, and BRD4 all reduce *Helicobacter pylori-*induced IL1B expression, but only the depletion of BRD4 significantly impairs *Helicobacter pylori-*induced IL1A expression.^96^

#### BRD4 regulates NFKBIA level

Depending on the context, BETs indirectly affect the activation of the RELA pathway by increasing or decreasing the expression of its upstream kinase IKBKB. For example, the deletion of BRD4 in macrophages results in the sustained expression of MAPK interacting serine/threonine kinase 2 (MKNK2) and activation of eukaryotic translation initiation factor 4E (EIF4E), which promotes the translation of *NFKBIA* mRNA, thereby reducing RELA-dependent inflammatory gene expression. Consequently, mice with myeloid lineage-specific deletion of the *Brd4* gene are resistant to LPS-induced septic shock and tissue injury.^97^ In contrast, in rheumatoid fibroblast-like synoviocytes stimulated by TNF, the knockdown of BRD4 or BRD2 reduces the phosphorylation and degradation of NFKBIA, resulting in the inactivation of RELA.^98^ It is still unclear which signal or checkpoint is required for BRD4-mediated up- or downregulation of NFKBIA.

#### STATs

The STAT family consists of seven members (STAT1, STAT2, STAT3, STAT4, STAT5A, STAT5B, and STAT6), which act as intracellular TFs in response to selective extracellular stimuli, such as cytokines (mainly IFNs and interleukins), growth factors, and PAMPs, by the membrane receptor-associated Janus kinase (JAK).^99^ The activation of the JAK-STAT pathway by phosphorylation leads to the production of cytokines and chemokines, thus affecting the immune response.

BETi shows strong activity in inhibiting the phosphorylation of JAK and STAT and subsequent transcriptional activity of STAT. For example, in a non-alcoholic steatohepatitis mouse model, I-BET151 treatment significantly reduced the expression of STAT1-dependent interferon gamma (IFNG) in liver tissue.^100^ Similarly, JQ1 suppresses genes enriched for a network of innate immune signaling nodes with a strong convergence on RELA-, JUN-, and STAT1-mediated transcriptional responses.^101^ I-BET 762 blocks LPS- and caerulein-induced phosphorylation of STAT3 in oncogenetic *Kras*^*G12D*^-driven mice, thereby blocking the production of nitric oxide and inflammatory cytokines (e.g., IL6, CCL2, and CSF2) in both immune and pancreatic cancer cells.^102^ I-BET 762 also decreases the phosphorylation of STAT3 in the mammary gland of MMTV-PyMT breast cancer mice, leading to the production of antitumor T-cell populations in mammary glands and spleen.^103^ These findings indicate the role of BETs in regulating the STAT3-dependent inflammatory tumor microenvironment and antitumor immunity.

More importantly, the BD2 domain of BRD2 recruits STAT3 to the chromatin through interaction with STAT3-K87Ac, thereby facilitating the recruitment of STAT3 to active enhancers occupied with interferon regulatory factor 4 (IRF4) and basic leucine zipper ATF-like transcription factor (BATF) and subsequent T helper (Th) 17 cell differentiation.^104,105^ In mouse embryonic fibroblasts, BRD4 is recruited to the STAT3-dependent suppressor of cytokine signaling 3 gene (SOCS3) and promotes its transcription.^106^ Like JQ1, the knockdown of BRD4 inhibits IL6-induced STAT3 activation and subsequent inflammatory gene production, thereby inhibiting the growth of pancreatic cancer.^35^ These findings further support the idea that BRD4-mediated STAT3 activation promotes tumor formation.

STAT5 mainly acts as a regulator of DC activation and is a crucial survival factor for NK cells. JQ1 can inhibit LPS-stimulated phosphorylation and nuclear accumulation of STAT5 in human monocyte-derived DCs, thereby decreasing STAT5’s transcriptional activity and impairing the maturation of monocyte-derived DCs.^107^ BRD2 is present at the transcriptionally active *Cis* locus and is required for the proper recruitment of STAT5-dependent transcriptional machinery for *Cis*.^108^ In IFNA1-induced monocytes, STAT1 and STAT2 are enriched on the promoter of IFN-responsive genes MX1 and CMPK2, and this process is inhibited by JQ1.^109^ Overall, these findings demonstrate that BETs mediate the activation of STAT pathways in various immune cells, which is highly related to infection, immunity, and tumorigenesis. Different STATs may exert overlapping and unique roles in BET-orchestrated immunity.

#### IRFs

IFRs are the main TFs responsible for antiviral immunity by producing IFNs. In small airway epithelial cells of humans infected with RSV, BRD4 recruits CDK9 to the promoters of IRF1, IRF7, and RIG, as well as to IRF3-dependent IFN-stimulated genes (ISGs), which in turn phosphorylates RNA Pol II at Ser 2 and enhances its expression through transcriptional elongation.^110^ Thus, BRD4 may play a protective role in the expression of airway mucosal IFN in response to RSV infection. BRD4 inhibition also induces host DNA damage response, thereby enhancing antiviral immunity by producting IFNs and ILs mediated by the CGAS-STING1-IRF3 signaling pathway.^39^ In LPS-induced bone marrow macrophages, the reduced expression of IRF4 and IRF8 mediates the effect of I-BET on suppressing the initial wave of inflammatory gene expression.^69^ Taken together, these findings highlight the importance of BRD4 in IRF-mediated antitumor immunity.

#### E2Fs

E2F is a family of TFs consisting of eight family members (E2F1 though 8). It regulates the cell cycle by repression or transactivation of genes that encode cyclins, cyclin-dependent kinases, checkpoint regulators, and replication proteins.^111^ In addition to the cell cycle, E2F is also involved in the inflammatory response, which is regulated by BETs. For example, E2F1 is citrullinated by peptidyl arginine deiminase 4 in inflammatory cells, thereby enhancing the binding of BRD4 to the acetylation domain in E2F1, resulting in the expression of proinflammatory genes.^112^ Though no direct evidence for the interaction between other BETs (BRD2 and BRD3) and the E2F family as well as their transcriptional regulation of immune genes has been reported, BRD2 is found to act as an E2F1- and H2A.Z variant histone 1 (H2AZ1)-interacting protein, which promotes the transcription of cell cycle-related genes.^112^ In addition, after JQ1 treatment, E2F3 shows enhanced motif activity, which may be related to the transcriptional regulation of BRD2.^113^ The details of BET-induced E2F activation still need further study.

#### MYC

The MYC family of proteins, a group of basic helix-loop-helix leucine zipper TFs that mainly coordinates cellular proliferation and metabolism, consists of four members: c-Myc (MYC, also known as c-MYC), L-Myc (MYCL), N-Myc (MYCN), and S-Myc.^114^ MYC (a BHLH transcription factor) is frequently altered in human cancers and promotes the transcription of various cell growth, apoptosis, and metabolism-related genes. Increasing evidence shows that MYC is an essential component for BET-regulated tumor immunity. For instance, JQ1 suppresses immune checkpoint CD274 expression by inhibiting the BRD4-MYC axis, indicating that the BRD4-MYC-CD274 pathway may mediate tumor immune escape.^115^ In neuroblastoma, JQ1 impairs the expression of ULBP1–3 ligands for NKG2D activating receptor by inhibiting the transcriptional regulation of MYC and tumor protein P53 (TP53), thereby rendering NB cell lines more resistant to NK cell-mediated killing.^116^ These studies demonstrate the dual role of BETs in antitumor immunity, depending on MYC status and cancer type.

#### Other TFs

In addition to the TFs discussed above, other TFs (e.g., TP53, RUNX2, SP7, JUN, FOS, ETS1, ETS2, CDX2, FOXL1, LIN54, RORC, ARIE, and FOXM1) also interact with BETs to participate in the transcriptional regulation of immune-related genes (Table 1 and Fig. 5). Studies further suggest that BETs are essential for TF-mediated gene transcription, although they may function in a context-dependent manner.

#### Transcriptional coactivators

Transcription activation is a complex, multi-stage process, involving by hundreds of proteins (including TFs and transcription coactivators). P-TEFb generally functions as a coactivator of BET-mediated gene transcription. It is composed of CDK9 and one of several other cyclin-related partners (e.g., cyclin T1), which release the paused RNA Pol II at the proximal promoter to allow transcription.^117^ Mechanistically, the BETs recruit CDK9 and cyclin T1 to the RNA Pol II,^20,118^ then activate it through phosphorylation of the C-terminal domains Ser2 and Ser5 for transcriptional extension.^119,120^ BRD4 can bridge autoimmune regulator (AIRE) and P-TEFb, thus promoting AIRE-mediated gene transcription in medullary epithelial cells and inducing effective immunologic tolerance.^121^ However, the molecular mechanism of transcriptional co-repressors and the way the protein can be converted from a coactivator to a co-repressor are unclear.

#### Histones

Binding to acetylated lysine residues on histone tails is a prerequisite for BET-mediated transcription activation. The core histones (H2A, H2B, H3, and H4) form the center of nucleosomes, which are linked by histone H1.^122^ BETs specifically recognize acetylated lysine residues in histone H3 and H4. Acetylation of lysine positions in the histone tail is performed by histone acetyltransferase enzymes (HATs). In most species, histone H3 is usually acetylated at lysine 9, 14, 18, 23, and 27, while histone H4 is mainly acetylated at lysine 5, 8, 12, and 16.^123^ IL1B or TNF induce the acetylation of H4K5Ac, H4K8Ac, and H4K12Ac, then recruits BRD3 and BRD4 to the promoter of the matrix degrading enzyme genes (MMP1, MMP3, MMP13, and ADAMTS4), thereby increasing their expression in human chondrosarcoma cell lines (SW1353).^118^ In virus-infected macrophages, BRD3 increases the acetylation of histone H3 and H4 within the IFNB1 promoter, leading to the production of type I IFN.^50^ Moreover, the accumulation of H4K5Ac and BRD4 on the IL6 gene promoter is found in lung fibroblasts from idiopathic pulmonary fibrosis donors, leading to increased IL6 production and secretion.^124^ In airway smooth muscle cells isolated from asthmatic individuals, histone H3 acetylation (especially H3K18Ac) increases, which helps BRD3 and BRD4 bind to the promoter of CXCL8 and promotes its expression, thereby driving steroid-resistant neutrophilic airway inflammation.^125^

Although BRD2, BRD3, and BRD4 are preferentially recruited to H4K5Ac, H4K12Ac, and H3K14Ac, H3K27Ac has attracted increasing attention in recent years. For example, investigators have found that BRD2, BRD3, and BRD4 directly bind to H3K27Ac at the promoter of 2, 3-dioxygenase 1 (IDO1) that mediates metabolism-related immune escape in cancer.^126^ The combination of BRD4 and H3K27Ac also facilitates the formation of SEs, which drives the transcription of NF-κB target genes (MT-CO2 and TGFB2), and then promotes the production of the extracellular matrix, myofibroblast differentiation, and tumor-associated inflammation.^127,128^ In addition, BETi (e.g., I-BET151) inhibits the deposition of H3K27Ac at the promoters of proinflammatory cytokines (TNF and IL6) induced by β-glucan.^129^ A global analysis of lysine acetylation may help us to better understand the function of BETs in gene transcription.

In the BET family, BRD4 acts as an atypical HAT, which can acetylate histone H3 and H4 in a different mode than other HATs.^128^ Because BRD4 can induce the acetylation of histone H3 on Lys residue 122 (H3K122Ac), which is a posttranslational modification that destabilizes nucleosome structure, the nuclear abundance of H3K122Ac is considered to be a selective marker for the HAT activity of BRD4.^15^ Both BRD4 and RELA are required for various stimuli (e.g., RSV, poly(I:C), and allergen)-induced acetylation of histone H3 on Lys 122, thereby promoting airway remodeling driven by inflammation.^55,58,110^ The functional interaction between BRD4 and classical HATs needs further clarification.

#### BETi in innate immunity

I-BET was discovered in 2010 and was the first BETi that was found to mimic acetylated histones to disrupt BET binding to chromatin. I-BET exhibits an anti-inflammatory effect on LPS-induced endotoxic shock and bacteria-induced sepsis.^69^ Since then, a large number of BETis (e.g., JQ1, I-BET151, OTX015, and I-BET762) have been developed, which exhibit excellent anti-inflammatory and immunomodulatory activities (Table 1). Unfortunately, due to the high structural homology in the two BD domains of BETs, most BETis are nonselective. Recently, selective inhibitors targeting BD1 and BD2 of the BET proteins have been developed, and it is proposed that BD1 is primarily required for steady-state gene expression whereas both BD1 and BD2 are required for the rapid inflammatory stimuli-induced increase of gene expression. As such, selective BD1 inhibitors phenocopy the effects of pan-BETi in cancer whereas selective BD2 inhibitors are dominantly effective in inflammatory and autoimmune disease.^130^ However, the long-term adverse reactions of these BETis are not clear. Hence, more selective BETis that target BD1 and BD2 with fewer toxic side effects are still in great need to achieve precise treatment of different diseases.

In the pathogenesis of various diseases, the roles of individual BETs overlap but are discrete, which indicates that it is still very important to develop new isotype-selective and well-tolerated BETis. In recent years, the degradation of BETs induced by proteolytic targeting chimera (PROTAC) has shown excellent targeting ability and inhibition. PROTAC-based BETis exhibit excellent immunoregulatory activities and include dBET1,^131^ MZ-1,^132^ and ARV-825.^95^ Importanly, dBET1 potently reduces proinflammatory responses in LPS-activated microglia by degrading BRD2 and BRD4, while ARV-825 increases NKG2D ligand MICA expression and sensitivity to NK cell-mediated cytotoxicity in multiple myeloma cells by degrading BRD4.^133^ MZ-1 redesigned based on JQ1 structure prevents TNF-induced expression of adhesion molecules and inflammatory mediators in the monocytes and endothelial cells by selectively degrading BRD4. These pan- or selective BETis provide a useful tool for studying the roles of BETs in regulating inflammation and immune response in vivo.

Of note, though proteolysis targeting chimera (PROTACs) have distinct advantages over small-molecule BETis, they may be limited to proteins that contain cytosolic domains to which ligands can bind and recruit the requisite cellular components. Lysosome-targeting chimeras (LYTACs) may be a more promising strategy to treat various diseases through selectively degrading BETs. These recently investigated chimeras use conjugates that bind both a cell surface lysosome-shuttling receptor and the extracellular domain of a target protein.^134^ In addition, phase-separated condensates that compartmentalize and concentrate anti-neoplastic drugs may facilitate LYTACs binding to BRD4 and have selective effects on oncogenes,^135^ although the efficiency of this approach is still controversial. Elucidating the three-dimensional structure and complexity of BETs is expected to allow us to obtain more effective and specific BETis.

### BETs in diseases

Because of their critical roles in the transcriptional regulation of genes, BETs have become promising therapeutic targets for various diseases (especially inflammation-related diseases, cancers, and metabolic diseases), as described below.

### Infectious diseases

#### Bacteria infection

Bacteria are the most common organisms that cause local and systemic inflammation, even sepsis and septic shock. LPS, a major component of the outer membrane of Gram-negative bacteria, is widely recognized as a strong activator of innate immune response. Both the genetic and pharmacological inhibition of BETs inhibit LPS-induced systemic inflammation or organ-specific inflammatory responses. For example, pan-BETi protects against endotoxic shock, polymicrobial peritonitis, polymicrobial sepsis induced by cecal ligation and puncture,^69^ LPS-induced periodontitis,^95^ and vascular inflammation.^13^ The genetic inhibition of BRD4 decreases NOS2 expression and inflammation response in *Listeria monocytogenes*-induced macrophages in vitro.^94^ In vivo, mice with a myeloid lineage-specific deletion of Brd4 are more sensitive to group B *Streptococcus*-induced infection, but are resistant to LPS-induced endotoxic shock,^97^ indicating a different role of BRD4 in infection. BRD2 also drives LPS-stimulated neuroinflammation and alveolar inflammation.^40,46^ Apart from LPS, listeriolysin-O, a hemolysin produced by the bacterium *Listeria monocytogenes*, increases BRD4 expression in Kupffer cells, which may induce liver injury by promoting necroptosis, inflammation, and mitochondrial dysfunction.^136^ Whether the functional impairment of BETs is the main cause and prognostic determinant of common bacterial infections and subsequent multiple organ dysfunction syndrome (especially disseminated intravascular coagulation) remains to be determined.

#### Viral infection

Viral infections have similarities and differences with bacterial infections. Most viral infections can be prevented by the innate immune system, and when the virus replicates beyond the innate defense, the adaptive immune response can be mobilized. As mentioned earlier, BRD4 inhibition enhances innate immune response, resulting in the inhibition of the attachment of DNA and RNA viruses through the CGAS-STING1 pathway.^39^ RSV replication increases the expression and binding of BRD4 to RELA, thereby triggering the inflammatory response in the lower respiratory tract and promoting airway remodeling.^58,110^ Moreover, treatment with pan-BETi also dominantly induces the resistance to influenza A virus (H1N1 subtype; strain WSN/33) by enhancing innate immunity.^94^ The inhibition of BETs leads to HIV promoter activation through separate modes of action, which may be beneficial for a combined anti-retroviral therapy.^137^ These findings generally suggest that increased BRD4 may be detrimental to antiviral immunity.

Conversely, BETs also suppress viral infection by maintaining IFNB production. Indeed, virus infection, such as through sendai virus, vesicular stomatitis virus, and herpes simplex virus, remarkably downregulate BRD3 expression in macrophages, thereby inhibiting the production of IFNB.^50^ As such, BET expression level is important for the establishment of antiviral immune homeostasis, and it may be useful for disease severity assessment and prognostic prediction of viral infection.

#### Fungi and parasitic infection

Invasive fungal infections usually result in high morbidity and mortality among immunocompromised individuals. The fungal BET protein BDF1, a global transcriptional regulator in *Saccharomyces cerevisiae*, also harbors two BD domains that are essential for the viability and virulence of *C. albicans*. Hence, BDF1 is proposed as a drug target for antifungal therapy.^138^ Although the exact mechanism between fungal infection, BETs, and host immune response has not yet been established, emerging evidence suggests that BETs (e.g., BRD2 and BRD4) may be involved in the differentiation of Th17 cells that can protect mucosa from bacterial and fungal infection through producing interleukin-17A (IL17A) and IL-17F.^104,139^ I-BET151 also inhibits the functions of human monocytes, lymphocytes, and granulocytes as well as the production of proinflammatory cytokines after *candida albicans* and *aspergillus fumigatus* stimulation,^129^ indicating an integrated immune modulation after BET inhibition.

Although the exact roles of BETs in parasitic infections are still elusive, BRD2 increases in livers infected with *Plasmodium yoelii* and *Toxoplasma gondii* and participates in host immune responses related to MHC class II, indicating a role of BRD2 in linking innate and adaptive immunity.^140^ In the mouse model of schistosomiasis, BET inhibition displays a protective effect on liver fibrosis by attenuating EP300-mediated RAR-related orphan receptor C (RORC) acetylation, thus decreasing the expression of RORC target genes.^141^ These findings indicate that BET, especially BRD2, predominant orchestrates innate immunity to fungi and parasitic infection, and it may serve as a potential therapeutic target for fungal and parasitic infections.

#### Non-infectious diseases

In addition to mediating infection, DAMPs can also initiate sterile inflammation, which is not only essential for tissue repair and regeneration, but also results in the development of numerous inflammation-related diseases, such as cancer, cardiovascular diseases, and metabolic disorders.^142^ As with PAMPs, accumulating evidence suggests the potential roles of BETs in controlling DAMP-mediated innate immunity and diseases.

#### Cancers

The development of cancer is a multi-step process and includes internal and external causes. It has been widely accepted that BETs-mediated transcription of pro-proliferative genes and anti-apoptotic genes plays a critical role in tumorigenesis. BETs inhibition down-regulates key oncogenic transcription factor pathways, thereby providing anti-tumor activity.^143–145^ Furthermore, BET-mediated innate and adaptive immune responses contribute to the development of cancers through the regulation of multiple events in the tumor microenvironment (TME), especially chronic inflammation and immune surveillance.

#### Chronic inflammation

The TME is a key factor in tumor progression and has been increasingly regarded as an anticancer therapeutic target. BETs regulate the TME through activating the transcription of proinflammatory genes in immune cell subsets infiltrating the TME. For instance, BRD4 can bind to the promoters of ARGINASE1 and other IL4-driven macrophage genes such as IL6, RETNLB, and CHIA, which results in immunosuppression in the TME.^146^ BRD4, but not BRD2 or BRD3, promotes TME inflammation in triple-negative breast cancer through upregulating multiple genes involved in extracellular matrix regulation, such as COL1A2, COL3A1, COL5A2, and KRT19.^147^ Chronic inflammation can induce DNA damage and accelerate gene mutations, which contribute to the development and progression of cancer. Like immune cells, cancer cells have the ability to release cytokines or chemokines to trigger tumorigenesis and metastasis. This process is called cancer cell-intrinsic inflammation and can be regulated by certain BETs. For example, BRD4 inhibition decreases CXC chemokine expression in clear cell renal cell carcinoma cells, resulting in a weakened inflammatory response and a reduced metastatic cascade.^148^ BRD4-mediated expression of IL6, RORC, COX2, MYC, CCND1, and CD47 also promotes the development of various solid cancers, such as pancreatic ductal adenocarcinoma, breast cancer, colorectal cancer, and myeloproliferative neoplasms.^35^ This inflammatory process is mainly driven by a BRD4-dependent activation of the NF-κB pathway in cancer cells.^61^ Thus, blocking the BRD4-NF-κB pathway may limit the development of cancer cell-intrinsic inflammation.

#### Immune surveillance

Through immune surveillance, the innate immune system plays a critical role in recognizing and eliminating tumor cells. This process is coordinated by various cells and proteins in the TME. In general, BETs may facilitate tumorigenesis via evading immune surveillance. For example, BRD4 expression is substantially upregulated in lymph node-negative breast cancer with a high expression of T-box transcription factor 21 (TBX21), which finally attenuates immune surveillance by upregulating jagged canonical notch ligand 1 (JAG1) expression.^149^ Oncogene-induced senescence is regarded as a potent barrier to tumorigenesis due to the generation of senescence-associated secretory phenotype (SASP). BRD4 is recruited to newly activated SEs adjacent to key SASP-related genes and mediates downstream paracrine signaling, thereby contributing to tumor-suppressive immune surveillance.^150^ Therefore, changes in the BRD4 pathway allow tumors to evade immune surveillance and to excessively proliferate, leading to their invasion into surrounding tissue structures and eventual metastasis.

Tumor antigen-specific cytotoxic T lymphocytes (CTLs) are the main effector of antitumor immune response. BETs also alter T-cell expression and function within the TME. For example, the T-cell population in the TME is changed by pan-BETi (I-BET 762) in some cancers.^103^ Personalized cancer vaccine is a cancer vaccine developed by encapsulating JQ1 and indocyanine green co-loaded tumor cells with a hydrogel matrix. This vaccine suppresses tumor relapse via promoting the maturation of DCs and eliciting of tumor infiltration of CTLs.^151^ JQ1 also has the ability to restore an immune-active environment by increasing intratumor DCs and CD8C T lymphocytes, and decreasing myeloid-derived suppressor cells.^34^ In addition, BETi enhances the immunogenicity of prostate cancer cells and the susceptibility to CD8 T-cell targeting by increasing MHC I expression.^152^

Immune checkpoints are usually activated in cancers to hinder the nascent antitumor immune response and become promising targets for cancer therapy. The CD274 checkpoint is transcriptionally regulated by histone acetyltransferase 1 in pancreatic cancer, and BRD4 is required for this process.^153^ Alternatively, BRD4 directly bind to CD274 promoter and regulates its transcription in pancreatic stellate cells.^154^ JQ1 greatly inhibits the expression of CD274, thereby overcoming the immunosuppressive effects in prostate cancer, lung cancer, and triple-negative breast cancer during cancer therapy.^115,152,155^ In addition, for cancers with relatively low responsiveness to immune checkpoint blockade therapy, I-BET762 enhances the efficacy of CD274 blockade by reducing the proportion of CD14 + HLA-DR/low myeloid-derived suppressor cells.^156^ In addition to BRD4, the gene status of BRD3 is also related to CD274 expression in cancer cells. In microsatellite high instability gastric cancer, the number of frameshift mutations in BRD3 is negatively correlated with CD274 expression.^48^ Apart from CD274, BETs also directly promote the expression of IDO1, an immune checkpoint that mediates metabolic immune escape in cancer through the production of L-kynurenine.^126^ Collectively, the evasion of immune surveillance by BETs covers multiple immunosuppressive mechanisms. The combination of BETi and traditional chemotherapy may restore or enhance antitumor immunity.

#### Cardiovascular diseases

DAMPs released from myocardial necrosis initiate inflammation to repair wounds and form scars. Nevertheless, persistent inflammatory response contributes greatly to myocardial remodeling and ultimately heart failure.^157^ BET-mediated transcription of proinflammatory and pro-fibrogenic genes are involved in various cardiovascular diseases, such as cardiac hypertrophy,^158^ pulmonary arterial hypertension,^62,159,160^ and myocardial infarction.^66^ The upregulation of BRD4 in cardiomyocytes may induce cardiac hypertrophy by increasing the expression of pro-fibrotic genes and activating RELA-driven inflammation.^158^ BRD4-mediated IL6 expression is increased in the coronary arteries, thereby promoting coronary artery remodeling during pulmonary arterial hypertension.^160^ Moreover, the binding of BRD4 to the SEs and promoters of proinflammatory or adhesion molecule genes in monocytes and endothelial cells promotes atherogenesis and the incidence of major adverse cardiac events.^63,132^ BRD4 may also promote the senescence and lipid accumulation in LPS-induced senescent macrophages by increasing the expression of SASP in autocrine and paracrine senescence, thereby promoting the progress of atherosclerosis.^161^ Accordingly, JQ1 exhibits therapeutic effects on heart failure caused by prolonged pressure overload and a massive anterior myocardial infarction.^66,101^ Similarly, BETi apabetalone (RVX-208) not only improves the cardiovascular outcomes of patients with type 2 diabetes after acute coronary syndrome,^162^ but also decreases the risk of atherosclerotic plaque rupture and major adverse cardiac events.^132^ These findings indicate that BETi can be used to treat a wide range of cardiovascular diseases, although its long-term side effects are currently unclear. Notably, the mechanisms of BETs in these cardiovascular diseases are far from being fully understood, and current studies mainly focus on the roles of BETs in the pathogenesis of chronic sterile inflammation-related cardiovascular diseases. A better understanding of the roles and mechanisms underlying BETs in sepsis-induced acute myocardial dysfunction, cardiomyogenesis, and myocardial regeneration after myocardial infarction should also be pursued in the future.

#### Respiratory diseases

Although infection is the leading cause of respiratory diseases, other pathogenic factors such as cigarette smoke extract and allergens, can also induce respiratory dysfunction. BRD4-mediated activation of the NF-κB pathway promotes lung inflammation, leading to airway remodeling in allergic airway disease.^163^ Moreover, BRD4-driven expression and secretion of IL6, CXCL8, and IL17A contribute to the development of idiopathic pulmonary fibrosis, chronic obstructive pulmonary disease, cystic fibrosis, and neutrophilic lung diseases.^26,27,101,140^ After growth factor stimulation, both BRD2 and BRD4 are involved in the regulation of filamentous ACTA2 expression in lung fibroblasts and drive pulmonary fibrosis.^164^ It remains to be seen whether BET-mediated production of proinflammatory cytokines affects immune cell infiltration and differentiation in infection-induced acute lung injury and various chronic lung diseases.

#### Neurological disorders

Sterile neuroinflammation is usually present in various central nervous system diseases, such as brain ischemia reperfusion injury, neurodegenerative diseases, and traumatic brain injury. The presynaptic neuronal protein α-synuclein is a pathological marker of Parkinson’s disease. It can trigger microglial activation by increasing BRD2 expression and subsequently inhibiting SIRT1 activation,^44^ indicating a pathologic role of BRD2 in Parkinson’s disease. In addition to Parkinson’s disease, dysfunctional BETs are also associated with Alzheimer’s disease due to their role in maintaining chronic inflammation.^165^ BETs promote systemic sclerosis by decreasing the acetylation and expression of two IFN-dependent genes (MX1 and CMPK2) in monocytes,^109^ suggesting that BET-dependent IFN signaling is a therapeutic target for systemic sclerosis. Moreover, the activation of BET pathway, especially BRD2-mediated inflammatory response and pyroptosis, aggravates cerebral ischemia-induced brain injury and acute spinal cord injury.^47,75,166^ Thus it can be concluded that BET-mediated activation of inflammatory pathways is an important pathological event leading to neurological disorders, and the correlation of BETs with other neuroinflammation-associated refractory diseases, such as depression, autistic disorder, and epilepsy, can also be attempted. In addition, as the immune system is closely interconnected with the state and function of the nervous system, research on the immunomodulatory roles of BETs expressed in different types of neurons is of great importance.

#### Kidney diseases

It is widely accepted that inflammation plays an important role in the pathogenesis of acute kidney dysfunction and chronic kidney diseases. Persistent low-grade inflammation is a hallmark of chronic kidney diseases and accelerates the loss of nephron functionality. BET-mediated expression of RELA-dependent proinflammatory genes (e.g., IL6, CCL2, and CCL5) and the activation of the Th17 immune response have considerable roles in acute renal damage caused by unilateral ureteral obstruction, lupus nephritis, and HIV-associated kidney disease.^95,167,168^ In addition, BET-mediated gene expression of pro-fibrotic factors promotes renal fibrosis and exacerbates renal dysfunction. Given that the pathophysiological process of kidney disease is complicated by various forms of ion pathway change, it is necessary to investigate whether BETs play a role in the disturbance of water and ion homeostasis as well as the endocrine function of the kidneys.

#### Digestive diseases

Given the role of BETs in the regulation of proinflammatory and pro-fibrotic gene expression, they are also involved in a diverse range of digestive diseases, such as non-alcoholic steatohepatitis and liver fibrosis,^100^ inflammatory bowel disease,^169^ acute pancreatitis,^170^ and colitis.^20^ Gut microbiota plays a critical role in the induction, training, and function of innate immunity, but their direct correlation with BETs has not yet been established. Regarding gut microbiomes and immunity, more comprehensive and in-depth research on BETs may expand our knowledge of and techniques against digestive diseases and improve clinical outcomes in the future.

#### Metabolic diseases

Metabolic abnormalities, such as diabetes mellitus, are also due to chronic low-grade inflammation. BRD2 is highly expressed in pancreatic β cells and physiologically inhibits INS transcription and β-cell mitosis. The knockdown of BRD2 results in severe obesity without type 2 diabetes mellitus, because BRD2 shifts energy balance toward storage without inducing glucose intolerance.^24^ However, this function of BRD2 in diabetes has been challenged by recent research. For example, the overexpression of BRD2 (but not BRD3 or BRD4) initiates chronic inflammation in adipocytes by activating RELA, thereby resulting in insulin resistance.^89^ These findings provide evidence that the upregulation of BRD2 may increase the susceptibility to type 2 diabetes.

BETs also contribute to the pathogenesis of type 1 diabetes mellitus. BETi (I-BET151) not only mitigates the immune response against pancreatic β cells, but also enhances their proliferation and function, thereby increasing insulin secretion and inhibiting the development of type 1 diabetes.^171^ Although BRD2 and BRD4 have the same activity to inhibit INS transcription, only BRD2 can inhibit fatty acid oxidation in β cells.^172^ Intriguingly, in *Drosophila melanogaster*, the BET protein *Fs(1)h* is required in fat body cells for a normal lifespan as well as metabolic and immune homeostasis. Flies lacking fat body fs(1)h exhibits a shorter lifespan, enhanced expression of immunotarget genes, disability to metabolize triglyceride, and systemic defects in insulin signaling.^173^ As mentioned above, the activation of the BRD4–NF-κB pathway contributes to the development of gouty arthritis, diabetic intervertebral disc degeneration, osteoarthritis, and rheumatoid arthritis, which have abnormal metabolic properties. In addition, both BRD2 and BRD4 play key roles in the onset of cancer cachexia by increasing the transcription of catabolic genes regulated by the IL6-AMPK-FOXO3 pathway.^174^ However, little is known about how BETs cause the interaction between immune and metabolic dysfunction in the TME, and how they link the metabolic disturbance of immune cells to immune dysfunction.

#### Osteoarthritis

Although osteoarthritis is traditionally regarded as a type of non-inflammatory arthritis, emerging evidence suggests that inflammation caused by DAMPs plays a vital role in the pathogenesis of osteoarthritis. As mentioned above, it has been proved that the BRD4-NF-κB signaling pathway has a great contribution to the development of gout arthritis, diabetic intervertebral disc degeneration, osteoarthritis, and rheumatoid arthritis.^51,74,98,175–177^

#### Other diseases

BETs are also involved in other diseases due to their immunomodulatory and proinflammatory properties, such as spontaneous preterm birth, preeclampsia, retinal inflammatory disease, inherited retinal degeneration, age-related macular degeneration, and psoriasis.^31,32,45,178–180^ It is expected that BETs may serve as potential therapeutic targets in a variety of immune-mediated diseases.

## Conclusion and perspectives

As epigenetic readers, BETs control the transcription of genes by recognizing acetylated histones and recruiting TFs and co-activators to the chromatin, thereby promoting the phosphorylation of RNA pol II and facilitating transcription initiation and elongation. BETs orchestrate various extracellular or intracellular danger signals through PRRs expressed in immune and non-immune cells in a wide range of diseases and have emerged as promising therapeutic targets. Although BRD4 is the most extensively studied member of the BET family, the exact role of other BET members (e.g., BRD2 and BRD3) in diseases and pathological conditions is still far from being fully understood. It is worth noting that a defect in BETs in mice causes embryo lethality, indicating that BETi may cause side effects. More studies are needed to develop isoform-selective and well-tolerated BETi or small-molecule PROTAC degraders and to clarify the distinctive roles of individual BETs in various cellular processes, including inflammation and immunity. High-efficiency and low-toxicity BETis may benefit the treatment of various immune-mediated inflammatory diseases in the future.

Although BETs play a crucial role in regulating gene transcription, the mechanism for this has not been fully elucidated. It is worth noting that BETs can recognize not only residues in histones, but also other acetylated nuclear proteins to control gene transcription.^181^ It can be supposed that other acetylation-regulated TFs, including cAMP response element binding protein 1 (CREB1), heat shock transcription factor 1 (HSF1), sterol regulatory element binding proteins (SREBPs), and carbohydrate response element binding protein (ChREBP), may also contribute to BET-orchestrated innate immunity.^182^ In addition to transcriptional regulatory activities, BETs also possess intrinsic kinase and lysine acetyltransferase (KAT) activities that have not been extensively studied.^15,22^ Hence, future research can also be extended to include the diverse transcriptional regulatory mechanisms of BETs and the roles of their kinase and KAT activities.

BETs play an emerging role in phase separation. The addition of BRD4 to acetylated chromatin can induce its liquid-liquid phase separation, so that different chromatin compartments can be established and maintained, thereby regulating gene transcription.^183^ The inherent disordered regions of BRD4 and MED1 can form phase-separated droplets, and then separate and concentrate the nuclear extract transcription device and control key cell-identity genes.^184^ In addition, LncRNA DIGIT can promote BRD3 to form a phase-separated condensate, which occupies the enhancer of endoderm transcription factor and drives the transcription of genes related to endoderm differentiation.^185^ These findings enhance the understanding of the pathological role of BETs in diseases and may identify new therapeutic targets.
